# Le syndrome de cushing chez l'adolescent: à propos de 18 patients

**DOI:** 10.11604/pamj.2015.22.347.8135

**Published:** 2015-12-11

**Authors:** Nassim Essabah Haraj, Siham El Aziz, Asma Chadli

**Affiliations:** 1 Service d'Endocrinologie Diabétologie et Maladies Métaboliques, CHU Ibn Rochd, Faculté de Médecine et de Pharmacie, Université Hassan II, Casablanca, Maroc

**Keywords:** Syndrome de cushing, adolescent, transition, Cushing syndrome, teenager, transition

## Abstract

Le syndrome de Cushing est une pathologie rare mais grave chez l'enfant et l'adolescent. Elle diffère de la pathologie adulte par le mode de présentation et la prise en charge. Il s'agit d'une étude rétrospective des dossiers de patients suivis pour syndrome de Cushing au service d'endocrinologie de Casablanca entre 2002 - 2015, incluant les patients âgés au moment du diagnostic de moins de 22 ans, et ayant un suivi d'au moins 1 an. Au total 18 dossiers ont été inclus. L’âge moyen est de 19,55 ans, avec une prédominance féminine. La durée d’évolution moyenne est de 4,05 ans. Le tableau clinique est fait souvent d'une cassure de poids, une obésité ou une séborrhée et acné. La démarche diagnostique est comparable à celle de l'adulte. Sur le plan étiologique on retrouve une prédominance de la maladie de Cushing (15 patients). Sur le plan thérapeutique, 14 patients ont bénéficié d'une chirurgie hypophysaire, avec complément par radiothérapie chez 3 patients devant l’échec de la chirurgie, Une ablation d'une tumeur surrénalienne chez une patiente et une surrénalectomie bilatérale chez trois patients. L’évolution a été marquée par une guérison chez 9 patients et le décès chez 4 (suite à un syndrome de Nelson, infection sévère, choc hémorragique, corticosurrénalome). Les résultats de cette étude soulignent la gravité de cette maladie, ce qui nécessite d'organiser le suivi, en élaborant des programmes spécifiques de suivi médical et de prise en charge psychologique.

## Introduction

Le syndrome de Cushing est une pathologie rare chez l'enfant et l'adolescent. L'incidence annuelle de la maladie de Cushing, qui est l’étiologie la plus fréquente chez l'adolescent, est de l'ordre de 1,2 à 1,7 nouveaux cas pour 1000 000 [1]. Le syndrome de Cushing chez l'enfant et l'adolescent diffère de la pathologie adulte par le mode de présentation et la prise en charge thérapeutique [2, 3]. Les moyens diagnostiques eux, sont relativement proches. Les sécrétions ectopiques d'ACTH ou les macroadénomes à ACTH sont exceptionnelles. Le début de l'adolescence est particulièrement difficile pour la plupart des jeunes, car c'est une période où ils se heurtent à divers problèmes associés à la transition entre l'adolescence et l’âge adulte. Chez les adolescents ayant un syndrome de Cushing cette période de transition clé présente des défis particuliers. L'un des défis majeurs est de juguler l'hypercorticisome pour conserver l'image du patient, d'assurer une croissance et une puberté normale et de répondre à leurs préoccupations de sexualité et de fertilité [1]. Cela implique une prise en charge multidisciplinaire, faisant appel à l'endocrinologue et l'endocrinologue pédiatre, puis au chirurgien et au psychiatre et psychologue. Nous nous sommes intéressés aux adolescents et jeunes adultes traités pour syndrome de Cushing, aux particularités cliniques, thérapeutiques et évolutives, au vécu et à l'impact psycho-social, afin d'approcher les difficultés de prise en charge au moment de l'adolescence et de la transition de cette pathologie lourde.

## Méthodes

Nous avons mené une étude rétrospective des dossiers de patients suivis pour syndrome de Cushing au service d'endocrinologie du CHU Ibn Rochd de Casablanca entre 2002 - 2015. Les patients inclus dans cette étude, répondaient aux critères suivants: âge au moment du diagnostic inférieur à 22 ans, et un suivi d'au moins 1 an après le diagnostic. Les données recueillies comprenaient: l’âge, le sexe, le motif de consultation, la durée d’évolution avant la consultation, les signes cliniques, les complications, le bilan biologique et morphologique, l’étiologie du syndrome de Cushing, le traitement (médical et chirurgical) et le suivi. Le recueil des données était fait par une fiche d'exploitation.

## Résultats

**Figure 1:**
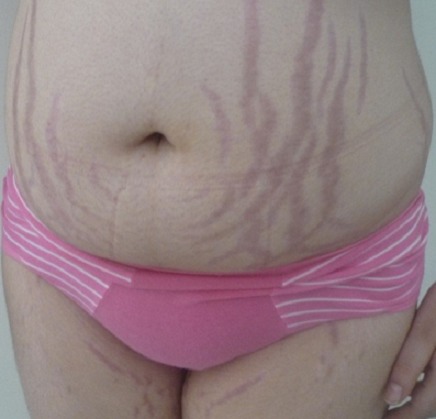
Obésité et vergetures abdominales chez une jeune adolescente avec maladie de cushing

**Figure 2:**
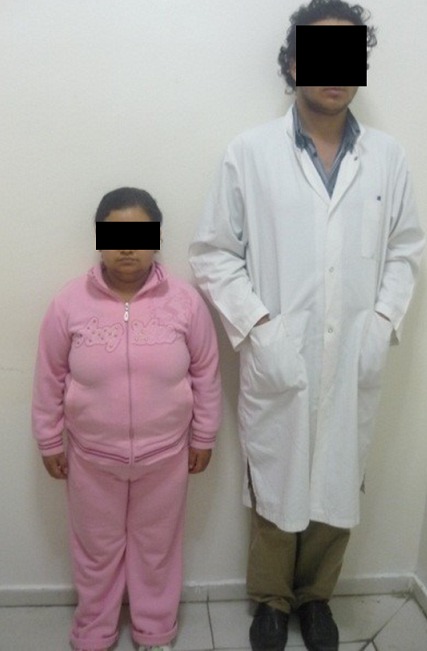
Retard staturo-pondéral et obésité chez une patiente avec maladie de Cushing

**Figure 3:**
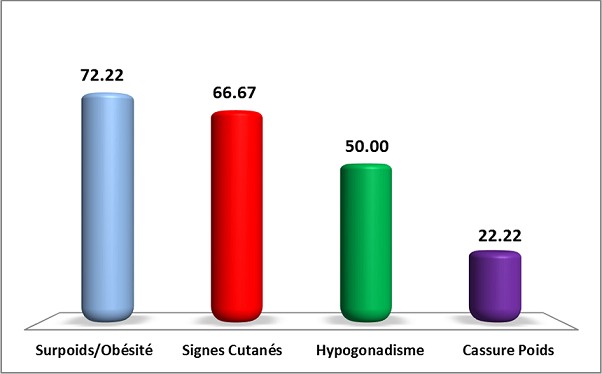
Signes cliniques retrouvés chez les adolescents avec un syndrome de Cushing

**Figure 4:**
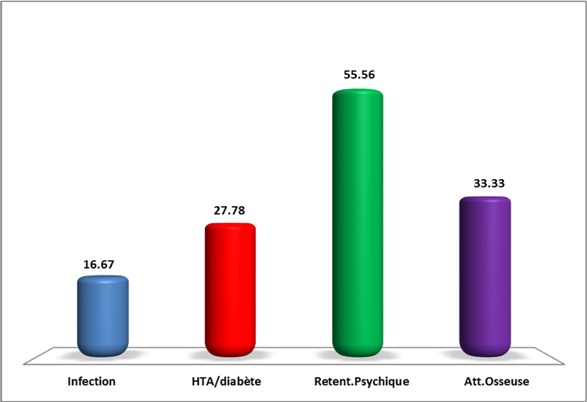
Complications retrouvées chez les adolescents avec un syndrome de Cushing

Au total 18 dossiers de patients ont été inclus dans l’étude. L’âge moyen des patients est de 19,55 ans avec des extrêmes de 14 ans à 22 ans. Une prédominance féminine a été retrouvée: 12 femmes/ 6 hommes. Et la durée d’évolution moyenne est de 4,05 ans avec des extrêmes de 1 à 12 ans. Le tableau clinique est en général moins typique et peut poser des difficultés de diagnostiques. Il est fait souvent d'une cassure de la courbe de poids, une obésité ou une séborrhée et acné. (Figure 1, Figure 2). La Figure 3 résume les différents signes cliniques présents chez nos patients. La démarche diagnostique est comparable à celle de l'adulte. Tous les patients ont bénéficié d'un dosage du cortisol libre urinaire et de l'ACTH. En fonction du bilan, une IRM hypophysaire ou TDM surrénalienne ont été réalisées. Un test de freinage fort a été réalisé chez 3 patients devant la suspicion d'un Cushing ectopique. Et une scintigraphie à l'octréotide a été réalisée chez un patient et s'est révélée normale. En ce qui concerne les complications de l'hypercorticisme (Figure 4): une infection sévère a été retrouvée chez 3 patients (un cas de tuberculose pulmonaire, un cas de pleuro-pneumopathie bactérienne extensive et un cas de furonculose des membres inférieurs). Une hypertension artérielle et un diabète étaient présents chez 5 patients. L'atteinte osseuse était retrouvée chez 6 patients, à type d'ostéoporose chez 4 et une fracture tassement chez 2 patients. Et les complications psychiques étaient majeures chez nos patients, retrouvés chez 10 patients, à type de dépression et anxiété, nécessitant un soutien psychologique et un traitement. Sur le plan étiologique, nous avons noté une prédominance du syndrome de Cushing ACTH dépendant (16 patients). Une maladie de Cushing a été retrouvée chez 15 patients (microadénome chez 10 patients, macroadénome chez 3 patients et une maladie de Cushing à IRM normal chez deux patients). Un corticosurrénalome a été retrouvé chez 2 patients. Et un syndrome de Cushing ectopique chez un patient avec absence de localisation de la masse.

**Figure 5:**
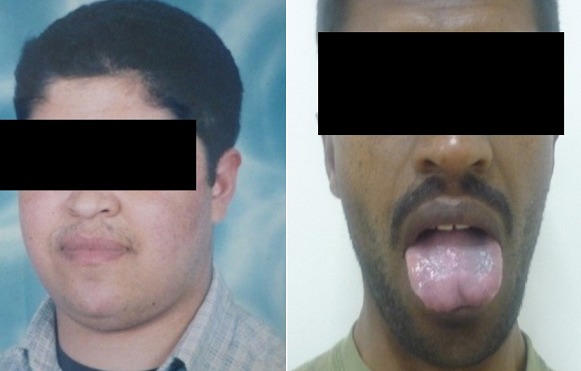
Syndrome de Nelson: patient avant et après la surrénalectomie bilateral

**Figure 6:**
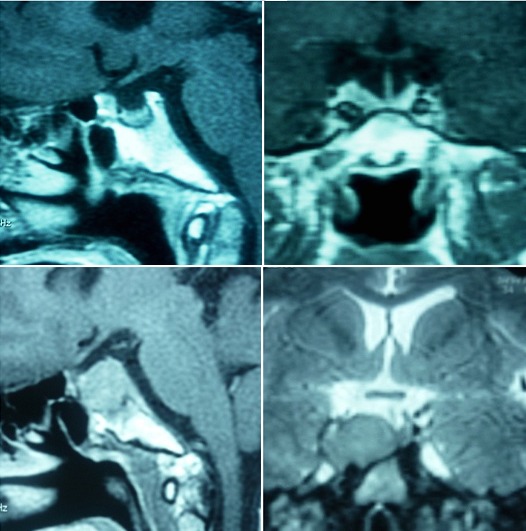
Syndrome de Nelson: IRM hypophysaire avant et après la surrénalectomie bilatérale montrant l'augmentation de la taille de l'adénome hypophysaire

Sur le plan thérapeutique, 14 patients ont bénéficié d'une chirurgie hypophysaire, avec complément par radiothérapie chez 3 patients devant l’échec de la chirurgie. Une ablation d'une tumeur surrénalienne a été réalisée chez une patiente. Une surrénalectomie bilatérale a été réalisée chez trois patients, devant un échec de chirurgie hypophysaire et radiothérapie chez un patient, devant une IRM normale avec aggravation clinique sous traitement médical chez une patiente, et un syndrome de Cushing ectopique sans individualisation de la tumeur chez un patient. Et 4 patients ont bénéficié d'une préparation médicale par du Kétoconazol avant la chirurgie. L’évolution a été marquée par une guérison après traitement chirurgical chez 8 patients, et suite à une apoplexie de l'adénome hypophysaire chez un patient. Un échec de la chirurgie chez 3 patients, nécessitant un complément de radiothérapie. Et le décès chez 4 patients: suite à un syndrome de Nelson post surrénalectomie bilatérale chez un patient (Figure 5 et Figure 6), un corticosurrénalome inopérable chez une patiente, à des complications infectieuses (pleuropneumopathie extensive) chez une patiente et à un choc hémorragique pendant la chirurgie chez une patiente. Trois patients ont été perdus de vu. Après guérison, on a obtenu une stabilisation des complications à type d'hypertension artérielle et de diabète chez tous les patients; une régression des signes cliniques cutanés et de l'obésité, et une stabilisation du retentissement osseux avec amélioration sous traitement, mais pas de reprise significative au niveau de la taille. Tous les patients ont bénéficié d'une exploration des autres axes de l'hypophyse et substitution du déficit. Concernant le retentissement psychosocial de la maladie chez nos patients, tous se sentaient différent des gens de leur âge, et déclaraient que les signes de leur maladie leur donnaient un sentiment de gêne, altérant leurs activités sociales (arrêt de scolarité chez deux patients). Tous rapportent une amélioration de leur qualité de vie après stabilisation de la maladie. Un patient rapporte qu'il est toujours limité par les douleurs osseuses surtout au niveau du dos. Deux patients trouvent qu'ils ont une petite taille par rapport aux gens de leur âge ce qui leur donne un sentiment de malaise. Une patiente s'interroge pour sa fertilité après la chirurgie hypophysaire et craint ne plus être en mesure d'avoir des enfants.

## Discussion

Le syndrome de Cushing est une pathologie grave par son retentissement physique et psychique et par ses complications. Sa gravité est encore plus importante durant la phase de transition. L'adolescent, n'ayant pas encore terminé sa croissance, est fragilisé sur le plan psychologique, soucieux de son aspect physique et de son avenir. Cette pathologie pose des particularités clinique et thérapeutique au cours de la phase de la transition [1]. Chez l'adolescent et l'enfant, on retrouve des difficultés diagnostiques. Le tableau clinique est souvent atypique, ce qui peut induire un retard diagnostique. Dans notre série, la durée moyenne d’évolution est en effet de 4,05 ans. Avec prédominance des signes à type de surpoids et d'obésité qui constituaient le motif principal de consultation. Les signes cutanés typiques du Cushing n’étaient pas toujours présents, et ils étaient retrouvés essentiellement chez les patients avec une longue durée d’évolution (supérieur à 3 ans). Le retard pubertaire et l'aménorrhée sont souvent associés dans ce cas, ceci a été confirmé dans notre série. Pour la diminution de la vitesse de croissance qui est toujours présente, dans notre série elle était retrouvée chez 4 patients. Ceci peut être expliqué par l’âge un peu avancé de nos patients, les patients inclus dans l’étude sont majoritairement de jeunes adultes, avec un âge moyen de 19 ans, ayant déjà terminé leur croissance. La déminéralisation osseuse est fréquente: elle est notée lors du diagnostic chez 75% des patients [2–5]. La démarche diagnostique dans le syndrome de Cushing chez l'adolescent est comparable à l'adulte [6]. La cause principale des syndromes de Cushing est la maladie de Cushing. Les tumeurs sont souvent des micro-adénomes. Les macro-adénomes sont rares. Le syndrome de Cushing ectopique est exceptionnel [1]. Ceci concorde avec les résultats de notre série: 15 patients avaient une maladie de Cushing. Les tumeurs surrénaliennes unilatérales sont majoritairement malignes chez l'enfant et l'adolescent. En effet chez les deux patients de la série ayant une masse surrénalienne il s'agissait de cortico-surrénalome. La présentation clinique des tumeurs surrénaliennes est différente. C'est la virilisation du patient qui fait évoquer le diagnostic plus que le syndrome de Cushing [5]. Le traitement du syndrome de Cushing chez l'adolescent a quelques particularités. Les enjeux de cette phase de transition sont la reprise de la croissance, le développement pubertaire, la fertilité ainsi que la restauration de la masse osseuse et la correction du syndrome métabolique [1, 7, 8]. Dans la maladie de Cushing, la chirurgie trans-sphénoïdale est le traitement de première intention. Le taux de guérison est globalement moins bon que chez l'adulte, avec une médiane de récidive plus courte [5]. L'insuffisance antéhypophysaire postopératoire est plus fréquente que chez l'adulte [1]. En cas de rechute ou d’échec de la chirurgie, un complément de traitement par radiothérapie peut être proposé. Les indications de la radiothérapie concernent essentiellement les adénomes invasifs [5]. Le traitement médical dans le syndrome de Cushing n'est pas recommandé au long cours. Il a des indications particulières, en cas d’échec ou de récidive, et d'emblée quand le micro adénome n'est pas visible [5, 9]. La surrénalectomie bilatérale doit être le dernier recours, vu le risque important du syndrome de Nelson. Le syndrome de Nelson chez l'enfant et l'adolescent reste grave et peut engager le pronostic vital [1]. En post chirurgical, un déficit en hormone de croissance peut survenir. Dans ce cas, le traitement substitutif est justifié. La recherche d'autres atteintes hypophysaires et l’évaluation du statut pubertaire sont nécessaires. Le risque de récidive du syndrome de Cushing est plus important chez l'enfant et l'adolescent, nécessitant une surveillance à long terme [5]. En ce qui concerne les complications et de leurs impacts psycho-sociaux à l'adolescence. Durant cette période, nos jeunes doivent effectuer une transition de développement, se forger une personnalité et une identité autonome et indépendante, en dehors de la famille. Cette transition peut être entravée par une image corporelle différente des autres suite à la maladie. En effet, nos résultats montrent la fragilité de nos patients qui restent affectés par la maladie. Tous ont exprimé le sentiment de gêne essentiellement par rapport à leur image. L'aspect physique et l'image corporelle prend une place importante, ce qui peut induire un comportement d'isolement rendant difficile leur insertion sociale. Le taux de dépression et d'anxiété était important dans notre série. Après traitement de la maladie toutes les séries notent une amélioration de la qualité de vie des patients mais celle-ci reste altérée par rapport à la population générale [10]. Nous nous sommes intéressés au vécu et à l'impact psycho-social des complications tout particulièrement à celle en rapport avec la puberté et la fertilité mais on remarque que ce sujet reste toujours difficile à aborder avec le médecin traitant. Une seule patiente a rapporté ses préoccupations par rapport à ce sujet. Cette étude montre que la prise en charge des jeunes adolescents atteints de Cushing reste assez hétérogène. Ce qui nécessite une prise en charge personnalisée, et adaptée à leur besoin. Le soutien psychologique est un pilier fondamental de la prise en charge de ces jeunes patients.

## Conclusion

Cette étude a mis l'accent sur les particularités du syndrome de Cushing chez l'adolescent et les difficultés rencontrées dans la prise en charge et le suivi de ces jeunes patients à la phase de transition. Il est donc important d'organiser le suivi de ces patients, en élaborant des programmes spécifiques de suivi médical, de prise en charge psychologique, et les programmes d'insertion sociale.
